# Effects of alcohol use on sperm chromatin structure, a retrospective analysis

**DOI:** 10.1186/s12610-023-00189-9

**Published:** 2023-06-08

**Authors:** Ariadne Trautman, Aarabhi Gurumoorthy, Keith A. Hansen

**Affiliations:** 1 University of South Dakota Sanford School of Medicine, Suite 2400, 409 Summit St, Yankton, SD USA; 2 Sanford Health, 2301 East 60th St N, Sioux Falls, SD 57104 USA; 3 Fertility and Reproductive Medicine Physician at Sanford Fertility & Reproductive Medicine, 1500 W 22Nd St Suite 102, Sioux Falls, SD 57105 USA

**Keywords:** Alcohol, Infertility, Sperm DNA fragmentation, Sperm chromatin structure analysis, Semen analysis, SCSA, Alcool, Infertilité, Fragmentation de l’ADN des Spermatozoïdes, Analyse de la Structure de la Chromatine des Spermatozoïdes, Analyse du Sperme, SCSA®

## Abstract

**Background:**

The evaluation of the infertile couple is often complex as multiple factors in both the male and female can contribute, including social history. Previous studies have displayed that male ethanol consumption can disturb sperm motility, nuclear maturity, and deoxyribonucleic acid (DNA) integrity. The main purpose of this study is to evaluate the effects of male alcohol use on sperm chromatin structure analysis (SCSA®).

This study was a retrospective chart review of 209 couples that presented to a midsize infertility clinic in the Midwest and had a semen analysis and SCSA® performed. Data extracted from the electronic medical record included demographics, tobacco use, alcohol use, occupational exposures, semen analysis results, and SCSA® results (DNA Fragmentation index (DFI) and High DNA stainability (HDS)). Statistical analysis was performed on this data set to determine significance with a p-level of 0.05, with the primary input being level of alcohol use and primary outcome being the SCSA® parameters.

**Results:**

Overall, 11% of the cohort had heavy alcohol use (> 10 drinks/week), 27% moderate (3–10/week), 34% rare (0.5- < 3/week), and 28% none. 36% of the cohort had HDS > 10% (a marker of immature sperm chromatin). Level of alcohol use was not significantly associated with HDS > 10% or DFI. Heavier alcohol use was significantly associated with lower sperm count (*p* = 0.042). Increasing age was significantly associated with increasing DNA Fragmentation Index (*p* = 0.006), increased sperm count (*p* = *0*.002), and lower semen volume (*p* = 0.022). Exposure to heat at work was significantly associated with lower semen volume (*p* = 0.042). Tobacco use was associated with lower sperm motility (*p* < 0.0001) and lower sperm count (*p* = 0.002).

**Conclusions:**

There was not a significant association between the level of alcohol use and the High DNA Stainability or DNA Fragmentation Index of sperm. Increasing age was associated with semen parameters as expected, heat exposure was associated with lower semen volume, and tobacco use was associated with lower sperm motility and density. Further studies could investigate alcohol use and reactive oxidative species in sperm.

**Supplementary Information:**

The online version contains supplementary material available at 10.1186/s12610-023-00189-9.

## Résumé

**Contexte:**

L’évaluation du couple infertile est souvent complexe car de multiples facteurs chez l’homme et la femme peuvent y contribuer, y compris l’histoire sociale. Des études antérieures ont montré que la consommation masculine d’éthanol pouvait altérer la mobilité des spermatozoïdes, la maturité nucléaire et l’intégrité de l’acide désoxyribonucléique (ADN). L’objectif principal de cette étude était d’évaluer les effets de la consommation d’alcool chez les hommes sur l’analyse de la structure de la chromatine des spermatozoïdes (SCSA®). Cette étude consistait en un examen rétrospectif des dossiers de 209 couples qui se sont présentés à une clinique d’infertilité de taille moyenne dans le Midwest et ont subi une analyse du sperme et un SCSA®. Les données extraites du dossier médical électronique comprenaient les données démographiques, le tabagisme, la consommation d’alcool, les expositions professionnelles, les résultats de l’analyse du sperme et les résultats du SCSA® (DFI et HDS). L’analyse statistique effectuée sur cet ensemble de données, pour déterminer la signification avec un niveau p de 0,05, a utilisé comme intrant principal le niveau de consommation d’alcool, le critère de jugement principal étant les paramètres du SCSA®.

**Résultats:**

Dans l’ensemble, 11% de la cohorte avait une forte consommation d’alcool (> 10 verres / semaine), 27% modérée (3–10/semaine), 34% rare (0,5 à < 3/semaine) et 28% aucune. 36% de la cohorte avait HDS > 10%. Le niveau de consommation d’alcool n’était pas significativement associé à un HDS > 10% ou au DFI. Une consommation d’alcool plus importante était significativement associée à une diminution du nombre de spermatozoïdes (*p *= 0,042). L’augmentation de l’âge était significativement associée à une augmentation de l’indice de fragmentation de l’ADN (*p* = 0,006), à une augmentation du nombre de spermatozoïdes (*p* = 0,002) et à une diminution du volume séminal (*p* = 0,022). L’exposition à la chaleur au travail était significativement associée à un volume séminal plus faible (*p* = 0,042). La consommation de tabac était associée à une mobilité plus faible des spermatozoïdes (*p* < 0,0001) et à une numération plus faible des spermatozoïdes (*p* = 0,002).

**Conclusions:**

Il n’y avait pas d’association significative entre le niveau de consommation d’alcool et la stabilité élevée de l’ADN ou l’indice de fragmentation de l’ADN des spermatozoïdes. L’augmentation de l’âge était associée aux paramètres du sperme comme attendu, l’exposition à la chaleur à un volume de sperme plus faible, et la consommation de tabac à une mobilité et une numération plus faibles des spermatozoïdes. Des études à venir pourraient explorer les relations entre consommation d’alcool et espèces oxydatives réactives dans le sperme.

## Background

The evaluation of the infertile couple is often complex, as multiple factors in both the male and female partner can contribute. Social history of the male is an important area that can sometimes be neglected when evaluating the couple. As spermatogenesis takes approximately 90 days, any detrimental event up to three months prior to attempted conception can affect sperm structure and function [1]. In particular, chronic alcohol use has been shown to have deleterious effects on spermatogenesis through multiple mechanisms [2]. Alcohol use has been connected with aberrations in testosterone metabolism as well as direct toxicity to the Leydig cells and the Sertoli cells [3–5]. Several studies have shown a lower percentage of morphologically normal spermatozoa in daily drinkers when compared to controls [6, 7]. Cytological aberrancies associated with alcohol use include coil-tailed sperm, immature testicular cells, breakage of the sperm head, and distention of the midsection [8, 9]. Heavy alcohol intake has also been associated with decline in sperm count and overall greater sexual dysfunction in men [1].

Many animal studies have been conducted to better characterize the effects of alcohol consumption on sperm and semen parameters. Investigation of the effects of ethanol consumption on chromatin condensation and DNA integrity of spermatozoa in rats revealed that ethanol consumption disturbed sperm motility, nuclear maturity, and DNA integrity, as well as produced sperm with less condensed chromatin [10]. A similar study on mice concluded that alcohol-induced sperm anomalies may be dose-dependent [11]. These mechanisms have also been assessed at a cellular level by intraperitoneal injection of ethanol in adult mice (3 g (15% solution)/kg body weight/day for 14 days) and subsequent evaluation of testicular androgenesis and germ cell apoptosis. Analysis by Western Blot revealed decreased expression of multiple enzymes involved in steroidogenesis and increased expression of apoptotic pathway-inducing enzymes [12].

Human studies on the effects of alcohol consumption on male fertility have analyzed these mechanisms at the pre-testicular (primarily hormonal axes), testicular, and post-testicular levels. Heavy alcohol consumers have been found to have significantly higher follicle-stimulating hormone (FSH), luteinizing hormone (LH), and estrogen levels compared to controls, with significantly decreased testosterone levels [6]. At the post-testicular level, several studies have investigated the correlations between male alcohol use and in vitro fertilization outcomes as well as implantation rates and pregnancy outcomes. A study looking at reproductive outcomes found that one additional alcoholic drink per day for men increased the risk of not achieving a live birth by 2.28 (1.08–4.80) to 8.32 (1.82–37.97) times, depending on the time period. It was thought the outcomes may be due to increased risk of miscarriage in couples where the male drank less than a month before and during in vitro fertilization (IVF) [13].

While research on hormone levels and semen parameters has been fairly extensive, other tools exist to specifically research sperm DNA chromatin structure, namely, the sperm chromatin structure analysis (SCSA®) test, which uses flow cytometry to measure sperm DNA fragmentation. Sperm DNA fragmentation is useful as a predictor of assisted reproductive technology (ART) success because it has been found to be associated with numerous fertility outcomes including fertilization, implantation, embryo development, miscarriage, and birth defects in the offspring [14]. The SCSA® was pioneered in 1980 by Donald Evenson and colleagues. The test measures two main factors, the DNA Fragmentation Index (DFI), which is a percentage of fragmented DNA in the sperm nucleus, and High DNA Stainability (HDS), which indicates the presence of immature sperm nuclei with abnormal proteins and/or altered protamine/histone ratios [15, 16]. Spermatozoa are stained with acridine orange, a dye that reveals broken DNA as red fluorescence and unbroken DNA strands as green fluorescence [17]. Studies have shown the %DFI level to be the best predictor for whether a couple will achieve pregnancy, with 25% becoming the accepted threshold above which the chance of viable pregnancy significantly decreases [15, 18]. A Danish study found that at %DFI > 20, male’s probability of fathering a child sharply declined [19]. Additionally, as HDS rises, it is associated with lower implantation rates and delayed/poor embryo development [20]. HDS > 15% has been associated with poor IVF fertilization rate [21].

A previous study focusing primarily on Vitamin D levels and SCSA® parameters found an incidental secondary result of elevated HDS in males who abstained from alcohol [22]. The present study aims to better evaluate the effects of male alcohol use on DNA Fragmentation Index and High DNA stainability. Investigation into the relationships between male alcohol use and SCSA® parameters will provide new information that will contribute to the knowledge base on the causes of infertility and will help physicians provide accurate information to their patients and better assist couples with understanding and treating infertility.

## Materials and methods

The study was conducted as a retrospective chart review of 209 consecutive couples who presented to a midsize infertility clinic in the Midwest and had a semen analysis and SCSA® performed between April 2017 and December 2020 as part of their evaluation. Semen samples were obtained by masturbation after 2–4 days of abstinence. The samples were ejaculated into a nontoxic specimen container and placed in a 37 °C water bath for 20–30 min for liquefaction. The semen analysis was performed manually after liquefaction by two trained technicians in the andrology laboratory using the 5^th^ edition of the World Health Organization (WHO) guidelines. [23]. The normal values of semen parameters used were semen volume of ≥ 1.5 mL, sperm count of ≥ 15 million/mL, total sperm motility of ≥ 40%, and level of spermatozoa with normal morphology of ≥ 4% using Kruger’s strict criteria [24]. Oligozoospermia was defined as < 15 million sperm per mL, asthenozoospermia was defined as a total sperm motility of < 40%, and teratozoospermia defined as < 4% normal forms by strict morphology.

A 0.2–0.5 mL sample of the raw semen was placed in a 1 mL cryovial and snap frozen in liquid nitrogen, then shipped to SCSA® Diagnostics for assay. The Sperm Chromatin Structure assay (SCSA®) is a highly precise and repeatable flow cytometry analysis used to measure acid-induced DNA fragmentation [15]. The semen sample is treated with acidic buffer solution (pH = 1.2) to allow the DNA to open at sites of DNA fragmentation and then treated with Acridine Orange (AO) staining solution composed of 0.20 M Na_2_HPO_4_, 0.1 M citric acid buffer (pH 6.0), 1 mM Ethylenediaminetetraacetic acid (EDTA), 0.15 M NaCl, and 6.0 ug/mL chromatographically purified AO. AO is a metachromatic dye that fluoresces green when associated with native, double-stranded DNA and red when associated with single-stranded DNA. After AO treatment, the sample is run through the flow cytometer where it is exposed to a 488 nm wavelength excitation beam from a 15–35 mW laser. Red (630–650 nm) and green (515–530 nm) filters collect the fluorescent signal from the excited, AO-stained sperm cells. An increase in red/green fluorescence is consistent with increased DNA fragmentation [17]. Parameters are collected based on red/green fluorescence intensity of the sperm sample. The raw data is sent to SCSAsoft® for analysis, which calculates the DNA Fragmentation Index (%DFI), moderately elevated %DFI, high %DFI, and %HDS. The %DFI is defined as the percent of sperm containing measurable DNA damage [15].

Prior to using the flow cytometer, alignment is determined using standard fluorescent beads. An AO buffer must pass through the instrument lines for at least 15 min prior to establishing settings with reference samples. The reference sample is chosen for heterogeneity of DNA integrity (eg. %DFI of around 15%) and is diluted to 1–2 million sperm/mL for use. All semen samples are assayed in duplicate with about 5,000 sperm cells in each measurement. Clinical Laboratory Improvement Amendments (CLIA) certification, which ensures quality laboratory testing in the United States of America, also requires reference samples with low and high %DFI be run for improved quality of analysis. During the use of the SCSA®, a fresh reference sample is run every 5 to 10 subject samples to exclude drift.

### Data acquisition

A query was run for patients who had an SCSA® performed to obtain the medical record numbers, and these charts were reviewed to determine male exposures and enter the data into a secure deidentified form. The data were entered independently into Excel spreadsheets by two investigators. The spreadsheets were then electronically compared in Excel and discrepancies resolved by reference to the electronic medical record (EMR) primary data. Inclusion criteria were male subjects who had an SCSA® performed and on whom alcohol use data was available. If data were not available on alcohol use, subjects were excluded. Of note, alcohol use was self-reported and the definition of what constitutes one alcoholic drink may have varied from subject to subject.

The data extracted and recorded included birthdate, race, past conceptions with current partner, past conceptions with previous partner, past and present infertility diagnoses for both partners, past infertility treatments, occupation, whether subjects worked primarily outdoors or indoors, alcoholic drinks consumed per week (split into beer, wine, and liquor), tobacco use (split into smoking and chewing), marijuana and illicit drug use, number of caffeinated drinks per day, exposure to radiation or chemicals (with exposures specified), exposure to heat (hot tubs, saunas, outdoor summer work, etc.), medical and surgical history, prescription medications, vitamins and supplements, family history, and date that SCSA® was performed. The outcomes recorded were the SCSA® results (DFI High (%), DFI Moderate (%), Total DFI (%), Mean DFI, Standard Deviation DFI, and HDS (%)) and the semen analysis results (semen volume (mL), sperm count (million sperm/mL), sperm motility (% motile), level of spermatozoa with normal morphology (%), Round Cells (RC) immature (million cells/mL), and RC Other (million cells/mL). Round Cells Immature and Round Cells Other were defined as according to WHO guidelines (5^th^ edition) [23].

### Statistical analyses

All analyses were performed in SAS 9.4. Continuous variables were summarized using n, means, standard deviation, minimum, and maximum. Categorical variables were summarized using frequencies and percentages. A sample size prediction calculation with a power level of 80% estimated that we needed at least 22 non-drinkers and 146 alcohol drinkers to achieve this power, criteria which was met by our sample.

Multivariable regression models were built to analyze the predictors for outcome variables. The primary outcomes analyzed were the SCSA® parameters of DFI High, DFI Moderate, Total DFI, and HDS. Sperm with moderate DFI typically have normal morphology, while those with High DFI have elongated nuclei and signs of apoptosis. Together, the percent of sperm with normal DFI, moderate DFI, and high DFI are added to obtain Total DFI [25, 26]. Based on the current body of research correlating Total DFI with pregnancy outcomes, DFI < 15% is considered “excellent to good”, DFI between 15 and 25% is considered “fair to good”, DFI between 25 and 40% is “fair to poor” and DFI over 40% is “very poor” [16].

The outcome of HDS was analyzed separately as a continuous variable using linear regression and as a binary variable defined as HDS > 10% and HDS ≤ 10% using a logistic regression model. The main predictor of interest was alcohol use. Alcohol use was measured as total drinks/week and was also divided into four categories: none, mild use (0.5- < 3 drinks/week), moderate use (3–10 drinks/week) and heavy use (> 10 drinks/week). The following secondary outcomes were introduced into the regression models: tobacco use (yes or no), heat exposures (yes or no), radiation or chemical exposure (yes or no), age (in years), and outside or inside occupation. Additional outcomes analyzed were sperm count, semen volume, and sperm motility. For all predictors and association between variables, differences were considered significant at *p* < 0.05.

## Results

Out of 210 subjects, 11% of the cohort had heavy alcohol consumption, 27% moderate, 34% mild, and 28% none. 21.5% of the cohort were positive for tobacco use. 27% of the cohort worked outside and 20% had regular exposure to heat. Mean alcoholic drinks per week was 3.6 (standard deviation 5.4 drinks). The mean age was 33 years (standard deviation 5.7 years) (Table 1).

**Table 1:** Demographics of Infertility Cohort. Shows the demographics and other relevant variables of our infertility cohort, composed of 209 human male subjects that presented with their female partners to a reproductive and fertility medicine clinic in the American Midwest. The table also includes the prevalence of different infertility diagnoses in the female partners

	**Subjects**	**Percentage**	**Mean**	**Minimum**	**Maximum**	**Standard Deviation**
**Age**			33.44	21	51	5.69
**Race**						
Caucasian	185	88.52%				
Asian	10	4.78%				
African American	9	4.31%				
Hispanic	4	1.91%				
Native American	1	0.48%				
**Current Partner Conceptions**						
0	124	59.33%				
1	41	19.62%				
> 1	44	21.05%				
**Past Partner Conceptions**						
0	184	88.04%				
1	15	7.18%				
> 1	10	4.78%				
**Infertility Diagnoses**						
Primary infertility	38	18.18%				
Secondary infertility	171	81.82%				
Female Partner:						
Irregular cycles	13	6.22%				
Endometriosis	17	8.13%				
Repeated Pregnancy Loss	26	12.44%				
Polycystic Ovarian Syndrome	68	32.54%				
Tubal disease	31	14.83%				
Decreased Ovarian Reserve	35	16.75%				
Advanced Maternal Age	33	15.79%				
Obesity and/or Diabetes Mellitus	38	18.18%				
Other Female Factor	20	9.57%				
Male Partner:						
Male factor	99	47.37%				
**Male Occupation**						
Outdoor	55	26.32%				
Indoor	150	71.77%				
No data	4	1.91%				
**Alcoholic drinks/week**			3.6	0	36	5.37
Beers/week			2.66	0	30	4.63
Wine/week			0.10	0	2	0.38
Hard Liquor/ week			0.84	0	21	2.35
**Alcohol Consumption Level**						
None	59	28.23%				
Rare	70	33.49%				
Moderate	57	27.27%				
Heavy	23	11.00%				
**Tobacco Use**						
Yes	45	21.53%				
No	164	78.47%				
**Average Caffeine Use**			1.85	0	12	1.66
**Heat Exposure**						
Yes	42	20.10%				
No	167	79.90%				
**Radiation/Chemicals Exposure**						
Yes	41	19.62%				
No	168	80.38%				
**Drug Use**						
Marijuana	3	1.44%				
Other (Cocaine)	1	0.48%				
None	205	98.09%				
**Past Medical History**						
None	88	42.11%				
Chronic Condition	121	57.89%				
**Family History**						
Infertility	18	8.61%				
Other	95	45.45%				
None	96	45.93%				

The primary outcomes associated with sperm chromatin integrity were HDS and DFI. 36% of the cohort had HDS > 10% (Table 2). Regression analysis of the input variable in relation to the binary outcome of HDS > 10% or HDS ≤ 10% did not show a significant relationship. Analysis was also done with the outcome of HDS as a continuous variable, with no significant correlation (Table 3).

**Table 2:** Semen Analysis and SCSA Outcomes. Shows the outcomes after semen analysis and sperm chromatin structure analysis on semen samples from our cohort of 209 human male subjects presenting with their partners to an infertility clinic in the American Midwest. The mean, minimums, maximums, and standard deviations were calculated in SAS 9.14

**Outcome**	**Mean**	**Minimum**	**Maximum**	**Standard Deviation**
Volume (mL)	3.32	0.6	7.1	1.41
Density (million/mL)	78.33	1	396	64.21
Motility (% motile)	62.11	5	85	13.48
Level of sperm with normal morphology (%)	4.68	< 1	14	2.15
RC immature (million/mL)	3.06	0	39	5.59
RC other (million/mL)	2.19	1.41	30	3.46
DFI TOTAL (%)	12.99	1.9	63	9.39
DFI MOD (%)	7.38	1.1	48.2	5.27
DFI HIGH (%)	5.63	0.7	37.6	5.01
Mean DFI	205.83	146.1	472.1	47.83
SD DFI	149.67	67	287.7	45.58
HDS (%)	9.71	1.5	41	5.83
	**Frequency**	**%**		
HDS > 10%	75	35.89		
HDS ≤ 10%	134	64.11		

*SCSA* Sperm Chromatin Structure Analysis, *RC* Round Cell, *DFI* DNA Fragmentation Index, *MOD* moderate, *HDS* High DNA Stainability, *SD* Standard Deviation

**Table 3:** HDS Results of Infertility Cohort. Shows the High DNA Stainability (HDS) results of our cohort of 209 human male infertility subjects on the sperm chromatin structure analysis test performed on their semen samples. This HDS was analyzed as a continuous variable and as a binary (HDS over 10% or under or equal to 10%). This was done as a logistics regression model for the categorical variables and a linear regression model for the continuous variables. No significant relationships were found

**Outcome: HDS > 10% or HDS ≤ 10%**					
**Variable**	**Value**	**Estimate**	**Standard error**	**Wald Chi-Square**	***p*-value**
Age	age in years	-0.05	0.03	2.59	0.1077
Exposure to heat (yes/no)	yes	-0.06	0.19	0.09	0.7591
Outside/inside job	inside	4.09	1.82	0.00	0.9821
Outside/inside job	outside	4.26	1.82	0.00	0.9813
Exposure to radiation/chemicals (yes/no)	yes	0.04	0.20	0.05	0.8281
Tobacco use (yes/no)	yes	0.25	0.18	1.89	0.1687
Level of alcohol use	heavy	-0.04	0.36	0.01	0.9121
Level of alcohol use	moderate	0.29	0.25	1.31	0.2519
Level of alcohol use	rare	-0.27	0.25	1.10	0.2951
**Outcome: HDS (%) continuous variable**					
**Variable**	**Value**	**Estimate**	**Standard error**	**t-value**	***p*-value**
Age	age in years	-0.10	0.08	-1.29	0.1985
Exposure to heat (yes/no)	yes	1.03	1.08	0.96	0.3406
Outside/inside job	inside	4.08	4.20	0.97	0.3315
Outside/inside job	outside	3.00	4.28	0.70	0.4845
Exposure to radiation/chemicals (yes/no)	yes	1.13	1.11	1.02	0.3108
Tobacco use (yes/no)	yes	1.83	1.01	1.81	0.0726
Level of alcohol use	heavy	-1.09	1.44	-0.76	0.4500
Level of alcohol use	moderate	0.77	1.10	0.70	0.4855
Level of alcohol use	rare	0.01	1.07	0.01	0.9892

*HDS* High DNA Stainability

**Fig. 1:**
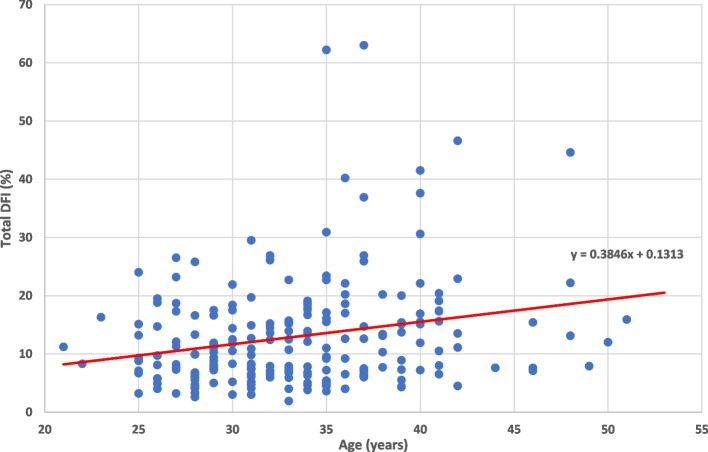
Shows a positive correlation between increasing age and increasing Total DNA Fragmentation Index (DFI) in our cohort of 209 human male subjects presenting with their partners to an infertility clinic in the American Midwest. A linear regression was performed to find the equation written above the trend line: “y = 0.3846 + 0.1313”. The *p*-value for this correlation was 0.0056

**Fig. 2:**
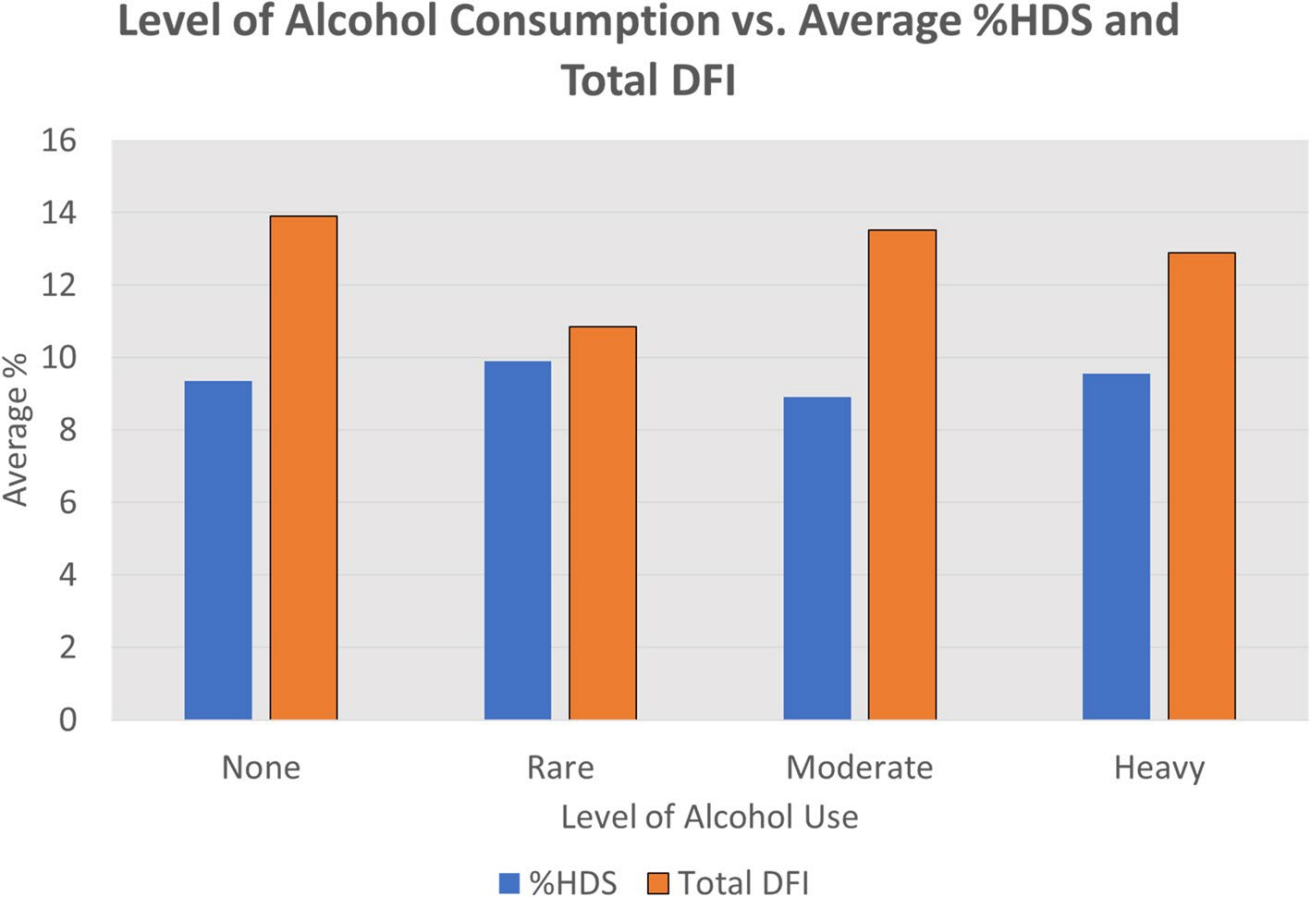
Breaks the alcohol consumption of 209 human male infertility subjects into four categories: none (0 drinks/week), rare (0.5- < 3 drinks/week), moderate (3.5–10 drinks/week), and heavy (> 10 drinks/week). The bars on the left denote the average %HDS (High DNA Stainability) of the men in each category and the bars on the right denote the average Total DFI (DNA Fragmentation Index) of the men in each category on a sperm chromatin analysis test

**Fig. 3:**
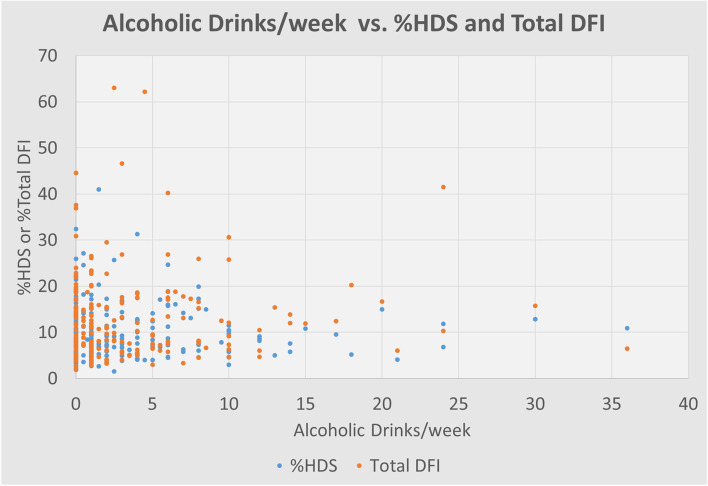
Compares self-reported alcoholic drinks/week with %HDS (High DNA Stainability) and Total DFI(DNA Fragmentation Index) on a sperm chromatin structure analysis in a cohort of 209 human male subjects presenting with their partners to an infertility clinic in the American Midwest. Each point on the scatter plot represents one subject. Analysis with linear regression did not reveal a significant relationship between the variables

The input variables were analyzed for their relationship to DFI High, DFI Moderate, and Total DFI. Average Total DFI in the cohort was 12.99% (Table 2). Increasing age had a significantly positive correlation with total DNA Fragmentation Index (*p* = 0.0056) (Fig. 1). Increasing age was also positively correlated with high DFI (*p* = 0.0037) moderate DFI (*p* = 0.039). Alcohol use did not have any significant correlation to HDS or DFI (Table 4; Figs. 2 and 3).

**Table 4:** DFI Results of Infertility Cohort. Shows the DNA Fragmentation Index (DFI) results of cohort of 209 human male infertility subjects as compared to multiple different lifestyle variables. Logistics regression models were used to test the relationship between categorical variables and DFI, and linear regression models were used to find the relationship between continuous variables and DFI

**Outcome: DFI High**					
**Variable**	**Value**	**Estimate**	**Standard error**	**t-value**	***p*-value**
Age	age in years	0.19	0.06	2.94	*0.0037
Exposure to heat (yes/no)	yes	0.42	0.91	0.46	0.6493
Outside/inside job	inside	2.70	3.55	0.76	0.4482
Outside/inside job	outside	1.18	3.62	0.33	0.7442
Exposure to radiation/chemicals (yes/no)	yes	0.02	0.94	0.02	0.9868
Tobacco use (yes/no)	yes	0.64	0.86	0.75	0.4559
Level of alcohol use	heavy	-0.26	1.22	-0.21	0.8308
Level of alcohol use	moderate	1.09	0.93	1.17	0.2428
Level of alcohol use	rare	-0.29	0.91	-0.32	0.7510
**Outcome: DFI Moderate**					
**Variable**	**Value**	**Estimate**	**Standard error**	**t-value**	***p*-value**
Age	age in years	0.15	0.07	2.17	*0.0309
Exposure to heat (yes/no)	yes	-0.09	0.98	-0.09	0.9252
Outside/inside job	inside	1.68	3.79	0.44	0.6577
Outside/inside job	outside	0.09	3.86	0.02	0.9823
Exposure to radiation/chemical (yes/no)	yes	0.16	1.01	0.16	0.8702
Tobacco use (yes/no)	yes	0.54	0.92	0.59	0.5577
Level of alcohol use	heavy	-1.22	2.65	-0.46	0.6460
Level of alcohol use	moderate	-1.31	1.25	-1.05	0.2962
Level of alcohol use	rare	-1.49	0.98	-1.51	0.1321
**Outcome: Total DFI**					
**Variable**	**Value**	**Estimate**	**Standard error**	**t-value**	***p*-value**
Age	age in years	0.34	0.12	2.80	*0.0056
Exposure to heat (yes/no)	yes	0.35	1.72	0.20	0.8394
Outside/inside job	inside	4.37	6.67	0.66	0.5130
Outside/inside job	outside	1.25	6.81	0.18	0.8544
Exposure to radiation/chemicals (yes/no)	yes	0.19	1.77	0.11	0.9139
Tobacco use (yes/no)	yes	1.19	1.61	0.74	0.4613
Level of alcohol use	heavy	-0.77	2.28	-0.34	0.7370
Level of alcohol use	moderate	0.02	1.75	0.01	0.9896

*DFI* DNA Fragmentation Index

^*^Denotes significance

**Fig. 4:**
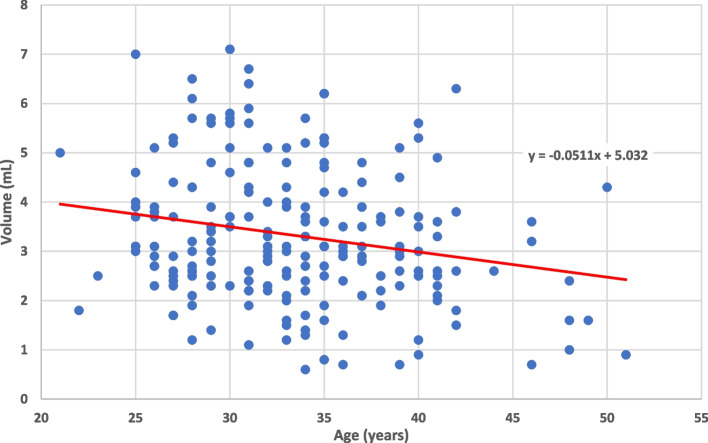
Plots the age of 209 human male infertility subjects against the volume (in milliliters) of their semen sample. Each dot represents a subject. The overall trend line was found through linear regression and shows a negative relationship between increasing age and semen volume (*p* = 0.0203)

**Fig. 5:**
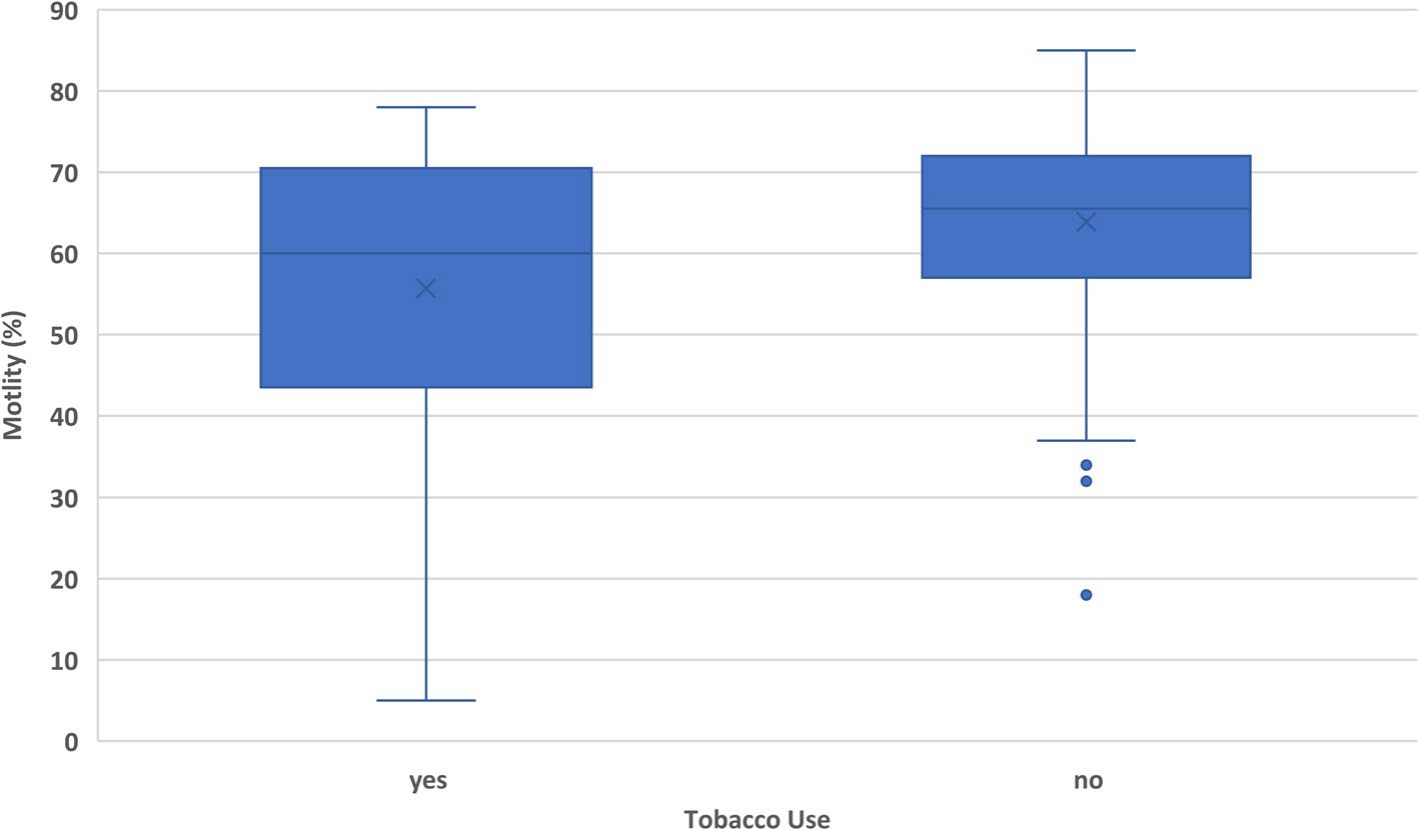
Shows the relationship between tobacco use and % motility of sperm (% motile sperm in a semen sample) in a cohort of 209 human male subjects presenting with their partners to an infertility clinic in the American Midwest. As can be seen, the mean and median motility are higher in non-smokers. There were 45 smokers and 164 non-smokers. This difference was significant when analyzed with a logistic regression model (*p* < 0.0001)

**Fig. 6:**
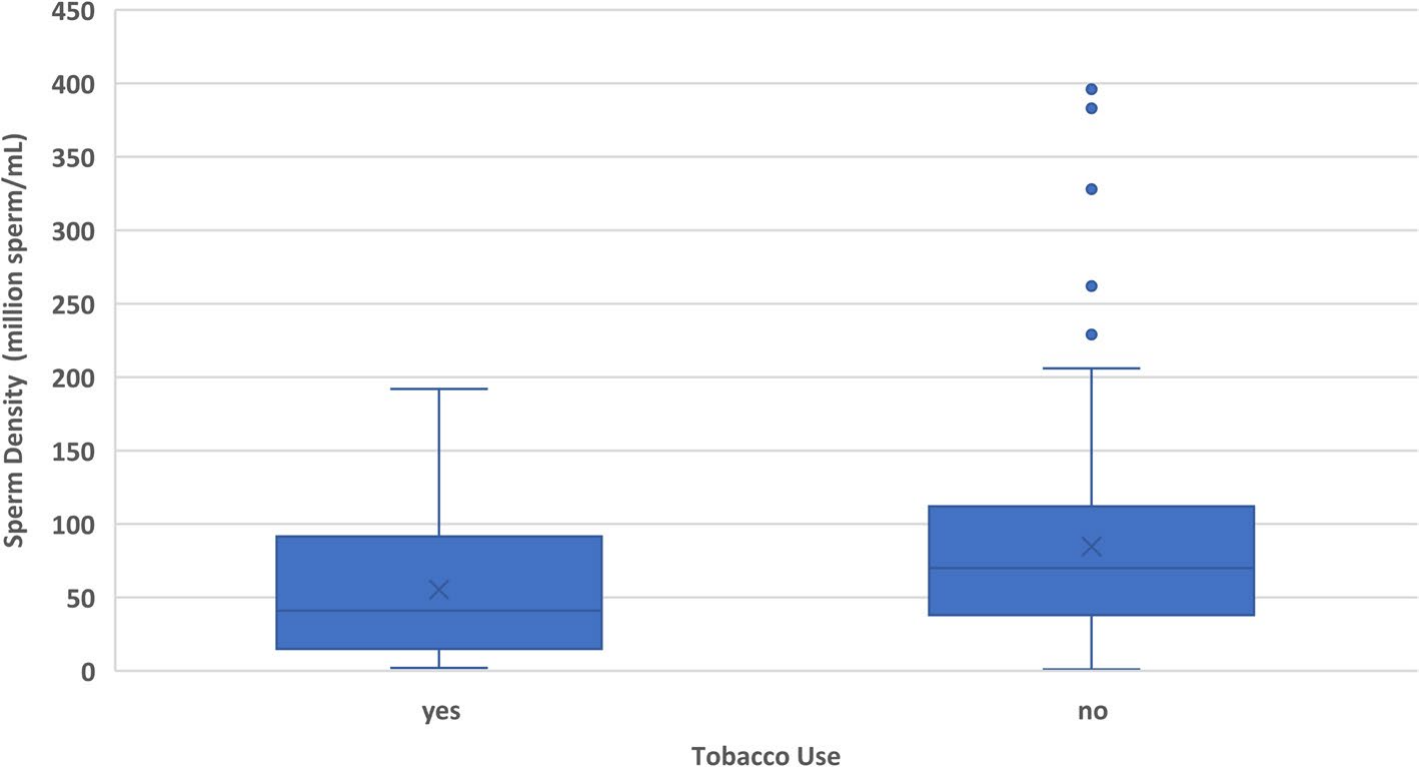
Shows the sperm count in a semen sample from 209 human male infertility subjects compared to their tobacco use status. There were 45 smokers and 164 non-smokers. The mean and median sperm count in non-smokers was higher. This difference was significant when tested with a logistic regression model (*p* = 0.0015)

**Fig. 7:**
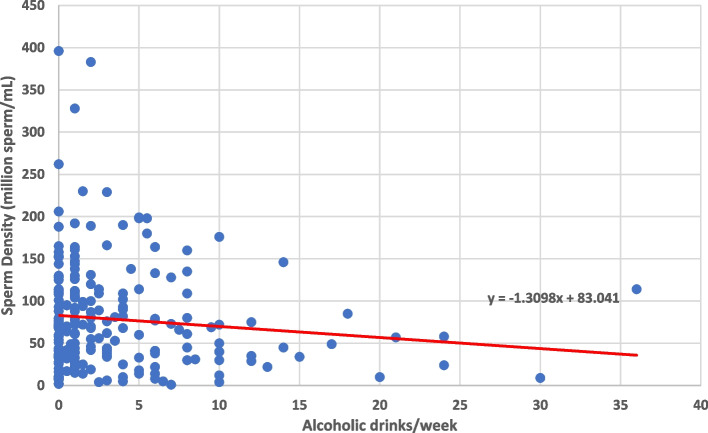
Shows sperm count in a semen sample plotted against self-reported alcoholic drinks/week in a cohort of 209 human male subjects presenting with their partners to an infertility clinic in the American Midwest. Increasing alcoholic drinks/week was associated with lower sperm count. This association was significant when tested with a linear regression model (*p* = 0.042)

In addition to the sperm chromatin structure values, the outcomes of sperm count, sperm motility, and semen volume were analyzed as well. Increased age was significantly correlated with increased sperm count (*p* = 0.0019) and decreased semen volume (*p* = 0.0203) (Fig. 4). Tobacco use had a negative correlation with sperm count (*p* = 0.0015) and a negative correlation with sperm motility (*p* < 0.0001) (Figs. 5 and 6). Heavier alcohol use had a negative relationship to sperm count (*p* = 0.042) (Fig. 7). Regular exposure to heat (hot tubs, saunas, outside summer jobs, etc.) had a negative correlation with semen volume (*p* = 0.0418). Outside occupation was associated with increased sperm motility (*p* = 0.0305) (Table 5). Supplementary files to the results include deidentified raw data from electronic medical records for all 209 subjects (Additional File 1) and full statistical analysis results (Additional File 2).

**Table 5:** Semen Analysis Results of Infertility Cohort. Shows the Semen Analysis results of our cohort of 209 human male infertility subjects compared to multiple lifestyle variables. Logistics regression models were run for categorical input variables and linear regression models were run for continuous input variables

**Outcome: Sperm Count (million sperm/mL)**					
**Variable**	**Value**	**Estimate**	**Standard error**	**t-value**	***p*-value**
Age	age in years	2.58	0.82	3.15	*0.0019
Exposure to heat (yes/no)	yes	9.87	11.54	0.85	0.3936
Outside/inside job	inside	36.03	44.87	0.80	0.4229
Outside/inside job	outside	38.91	45.75	0.85	0.3961
Exposure to radiation/chemicals (yes/no)	yes	14.76	11.92	1.24	0.2170
Tobacco use (yes/no)	yes	-34.99	10.85	-3.23	*0.0015
Level of alcohol use	heavy	-29.50	15.36	-1.92	*0.042
Level of alcohol use	moderate	0.93	11.73	0.08	0.9370
Level of alcohol use	rare	14.51	11.46	1.27	0.2071
**Outcome: Volume (mL)**					
**Variable**	**Value**	**Estimate**	**Standard error**	**t-value**	***p*-value**
Age	age in years	-0.04	0.02	-2.34	*0.0203
Exposure to heat (yes/no)	yes	-0.52	0.25	-2.05	*0.0418
Outside/inside job	inside	1.53	0.99	1.56	0.1212
Outside/inside job	outside	1.89	1.01	1.88	0.0610
Exposure to radiation/chemicals (yes/no)	yes	0.28	0.26	1.06	0.2922
Tobacco use (yes/no)	yes	-0.07	0.24	-0.30	0.7645
Level of alcohol use	heavy	0.27	0.34	0.80	0.4240
Level of alcohol use	moderate	0.42	0.26	1.64	0.1032
Level of alcohol use	rare	0.49	0.25	1.95	0.0529
**Outcome: Motility (%)**					
**Variable**	**Value**	**Estimate**	**Standard error**	**t-value**	***p*-value**
Age	age in years	-0.15	0.17	-0.89	0.3724
Exposure to heat (yes/no)	yes	0.38	2.40	0.16	0.8745
Outside/inside job	inside	16.28	9.33	1.74	0.0827
Outside/inside job	outside	20.74	9.52	2.18	*0.0305
Exposure to radiation/chemicals (yes/no)	yes	1.76	2.48	0.71	0.4797
Tobacco use (yes/no)	yes	-9.47	2.26	-4.19	* < 0.0001
Level of alcohol use	heavy	1.94	3.19	0.61	0.5451
Level of alcohol use	moderate	-0.47	2.44	-0.19	0.8476
Level of alcohol use	rare	2.06	2.38	0.86	0.3893

^*^Denotes significance

## Discussion

There was no significant correlation between the level of alcohol use and the HDS or DFI of sperm. This contradicts the previous study that found a higher HDS in non-drinkers [22]. The only significant association found to correlate with alcohol consumption was a lower sperm count with heavier alcohol use. The data were not controlled for confounding variables due to the relatively small sample size, so it was difficult to determine whether an isolated relationship might exist between alcohol consumption and sperm chromatin structure parameters. The decreased sperm count with heavy alcohol use was in line with prior studies finding a decrease in standard WHO semen analysis parameters in alcoholics [6, 8]. The primary source of DNA fragmentation in spermatozoa is oxidative damage [27]. A prior study measured oxidative stress (OS), enzymatic antioxidant activity (EAO), and DNA fragmentation of spermatozoa in four different groups of male infertility patients: non-alcohol and non-tobacco users, alcohol users, tobacco users, and alcohol and tobacco users. They found increased EAO and higher OS in the latter three groups, especially the tobacco and alcohol-tobacco groups. The highest level of DNA fragmentation and chromatin decondensation was present in the alcohol-tobacco group. This was in contrast with the lack of significant impact from alcohol use on the DNA fragmentation variables in our study [28].

There has been no definite consensus in prior literature on the effects of either alcohol or tobacco use on sperm parameters. One prior study found no significant effect of either alcohol or smoking on traditional semen parameters or pregnancy outcomes when studied in a group of sub-fertile men [29], while a more recent study comparing heavy drinkers to heavy smokers concluded that alcohol use deteriorated sperm maturity and damaged DNA integrity at “significantly higher rates” than tobacco use [30]. Other studies have found no association between alcoholic drinks per week and quality of sperm parameters or ultimate fertility outcome [31]. Interestingly, some previous research has found a protective effect on traditional semen parameters with moderate alcohol consumption, a so-called “U-shaped trend” thought to potentially stem from the antioxidant content of some kinds of alcohol [32, 33]. As in our study, most prior literature was conducted with subjects who were already infertility patients, complicating the range of potential causes of semen abnormalities and eventual fertility outcomes. A study with a larger stratified cohort with increased control for potentially confounding variables might offer a more definite answer.

This study did find significant effects of tobacco use on multiple semen parameters including lower sperm count and lower motility. A prior study on cigarette smoking found similar results, with a linear correlation between increased cigarette use and worsening motility and decreased chromatin condensation [34]. By contrast, another study found lower semen volume in cigarette smokers but no significant effects on other sperm parameters [35]. Other previous research has found cigarette smoking to be associated with a decrease in antioxidant enzyme activity but no increased oxidative DNA damage [36]. Overall, the data for the negative effect of cigarette smoking on semen parameters were somewhat more robust than for alcohol, which was reflected in our study as well [32].

One variable that significantly correlated with worsening sperm parameters in almost every category was age. This association was expected and is in accordance with the literature. With the SCSA® specifically as well as traditional semen analysis, age has previously been strongly correlated with increased DFI as well as decreased semen volume, decreased motility, and increased density [37]. Another study of over 25,000 males found similar results, with older men having increased %DFI and decreased %HDS (indicating more condensed chromatin) [18]. Our study showed age to be correlated with increased DFI, lower semen volume, and increased sperm count. There was also correlation between age and slightly decreased HDS and decreased motility, but not to a significant level. The final two findings were significant correlation between exposure to heat and lower semen volume and between outdoor occupations and higher sperm motility. Exposure to heat is well known for negative effects on semen analysis parameters. A prior study involved placing a wool sock over bull testes for 48 h and showed a doubling of %DFI with this [38]. The higher sperm motility in those with outdoor occupations was only a few percent higher than among those with indoor occupations and was likely incidental.

The implications of lifestyle factors potentially leading to DNA damage in sperm are numerous. Besides impact on fertility, there is also the consideration of possible transgenerational health effects from the damage. Any condition causing oxidative damage in spermatozoa has the potential to transmit damage to the offspring, possibly contributing to neuropsychiatric disorders, cancer, and a number of other potential effects [39]. The majority of de novo mutations in an embryo (around 75%) are known to originate in the paternal germ line [40]. Thus, modifying any lifestyle or environmental factors known to be associated with DNA damage could provide a solution to these concerns. The cessation of smoking in particular has been associated with improved sperm DNA parameters [41]. Abstinence from alcohol has been shown in several case studies to reverse azoospermia [42, 43]. Although this study has not found significant correlation between level of alcohol use and SCSA® parameters, it is worth noting that the average alcohol consumption in the study group was moderate and a study conducted with a larger proportion of chronic heavy drinkers might have revealed worsening SCSA® parameters to accompany the known semen abnormalities that heavy alcohol use is linked with. As both worsening DFI and increased alcohol consumption are associated with worsening IVF outcomes [13, 44], it would be reasonable to recommend at least a reduction if not cessation of alcohol consumption for both members of couples undergoing IVF or intracytoplasmic sperm injection (ICSI).

This study was limited by small sample size and selection bias as subjects were couples presenting for infertility treatment and were not representative of the population as a whole. There was also the possibility for confounding factors in the analyses. A future study with a larger cohort could control for variables like age, medical diagnoses, obesity, and tobacco use in order to study alcohol use individually. Other possible outcomes to measure in relation to alcohol use include hormone levels, oxidative damage, and antioxidant enzyme activity as well as more tangible outcomes such as rates of viable pregnancies, miscarriage, and livebirths. These outcomes could be measured in addition to the SCSA® parameters to analyze for correlation between the DNA fragmentation studies, hormone levels, oxidative damage, and infertility outcomes.

## Conclusions

Overall, while a significant association between alcohol consumption and sperm DNA fragmentation was not found in this study, heavier alcohol use was associated with lower sperm count. Additionally, tobacco use was associated with lower sperm count and lower motility. This research highlights the impact of social habits on sperm DNA structure, an important consideration in couples being evaluated and treated for infertility.

## Supplementary Information

**Additional file 1.**

**Additional file 2.**

## Data Availability

The dataset supporting the conclusions of this article is included within the article’s additional files. Additional File 1.xlsx (Microsoft Excel Workbook). Alcohol SCSA Data: original deidentified data collected from the EMR for 209 subjects. Additional File 2.xlsx (Microsoft Excel Workbook). Alcohol SCSA Analysis Results: raw statistical analysis results.

## Abbreviations

AO: Acridine Orange
ART: Assisted Reproductive Technology
CLIA: Clinical Laboratory Improvement Amendments
DFI: DNA Fragmentation Index
DNA: Deoxyribonucleic acid
EAO: Enzymatic Antioxidant Activity
EDTA: Ethylenediaminetetraacetic acid
EMR: Electronic Medical Record
FSH: Follicle-stimulating Hormone
HDS: High DNA Stainability
ICSI: Intracytoplasmic Sperm Injection
IVF: In Vitro Fertilization
LH: Luteinizing Hormone
OS: Oxidative Stress
RC: Round Cells
SCSA®: Sperm Chromatin Structure Analysis
WHO: World Health Organization
